# Supplementary material for: Metabolomic Profiling Reveals Distinct Signatures in Primary and Secondary Polycythemia
Source: Metabolites. 2025 Sep 22;15(9):630. doi: 10.3390/metabo15090630 (PMC12471915; doi:10.3390/metabo15090630)
Supplement: Supplementary file 1 [file metabolites-15-00630-s001.zip › Tables S1-S2.pdf]

Supplementary Table S1: Altered metabolites list of polycythemia vera vs secondary polycythemia (*p*<0.05, Fold change >1.5)

Peak no	Mass to charge	**Metabolite**	**HMDB ID**	**Retention time (min)**	*p* value	**Fold Change (+,-)**
1	94.9235	Thiocyanate	HMDB0001453	1.39	2.25E-06	1.90
2	96.9206	Hydrogen selenide	HMDB0011110	1.39	6.95E-07	1.98
3	97.9588	Hydrogen carbonate	HMDB0000595	2.62	4.28E-04	2.81
4	103.0385	Acetaldehyde	HMDB0000990	1.56	3.91E-03	1.63
5	115.0398	alpha-Ketoisovaleric acid	HMDB0000019	1.59	6.04E-05	-2.16
6	120.0115	L-Cysteine	HMDB0000574	6.62	5.52E-03	-1.72
7	132.0291	L-Aspartic acid	HMDB0000191	1.33	2.57E-06	3.60
8	135.0282	Glycolate	-	1.39	1.38E-04	1.66
9	137.0233	4-Hydroxybenzoic acid	HMDB0000500	3.48	1.17E-02	2.69
10	139.0384	Phenol	HMDB0000228	6.50	9.78E-04	-2.88
11	143.0792	Estriol	HMDB0000153	4.10	1.39E-03	-1.60
12	157.0942	4-Hydroxyretinoic acid	HMDB0006254	4.78	1.32E-08	-2.62
13	158.0449	Succinic acid	HMDB0000254	1.37	1.94E-03	2.34
14	164.0698	4-Guanidinobutanamide	METPA0359	2.52	3.08E-05	1.59
15	167.9611	Dichloroacetate	METPA0988	2.52	9.05E-03	3.39
16	183.9925	Cysteic acid	HMDB0002757	2.54	1.55E-03	6.46
17	187.0734	Glutamic acid	HMDB0000148	4.80	3.39E-05	-1.98
18	191.0180	Citric acid	HMDB0000094	1.39	2.37E-02	1.91
19	195.1007	Perillyl aldehyde	HMDB0003647	9.06	4.12E-02	-2.48
20	197.0642	D-Xylitol	HMDB0002917	1.41	2.82E-02	-1.51
21	203.0008	Pyridoxal	HMDB0001545	8.19	4.83E-02	-2.01

22	209.0522	Octanol	HMDB0001183	1.36	1.52E-03	-1.94
23	212.0234	3-Sulfinoalanine	HMDB0000996	2.63	1.93E-06	13.14
24	217.0289	D-Glucose	HMDB0000122	1.41	7.51E-06	4.29
25	224.0603	Dehydrospermidine	HMDB0012925	9.49	9.98E-03	-1.59
26	226.9747	(S)-2-Aceto-2-hydroxybutanoic acid	HMDB0006900	2.66	2.27E-05	2.20
27	232.9733	Nudifloramide	HMDB0004193	1.06	1.20E-07	-2.35
28	233.0755	Formiminoglutamic acid	HMDB0000854	6.11	1.62E-04	-1.95
29	237.0757	1,2-Dihydroxy-3,4-epoxy-1,2,3,4-tetrahydronaphthalene	HMDB0062273	6.57	9.46E-03	-2.21
30	241.0698	Homovanillic acid	HMDB0000118	6.41	1.70E-02	-2.35
31	256.0123	Citrulline	HMDB0000904	6.77	5.29E-04	-1.60
32	260.0218	L-3-Hydroxykynurenine	HMDB0011631	1.44	3.92E-03	1.58
33	264.1015	5-Hydroxykynurenine	HMDB0012819	5.96	3.39E-02	2.04
34	268.7983	Iodine	HMDB0000675	1.41	5.46E-06	2.77
35	269.0730	N2-Succinyl-L-ornithine	HMDB0001199	3.84	3.34E-02	-1.51
36	273.0697	Deoxyuridine	HMDB0000012	5.10	2.47E-11	-2.71
37	313.0619	Geranyl-PP	HMDB0001285	4.77	3.10E-02	-1.82
38	314.0648	D-4'-Phosphopantothenate	HMDB0001016	4.50	3.73E-05	-2.09
39	315.0389	Nicotinamide ribotide	HMDB0000229	5.22	2.67E-03	-2.06
40	316.0424	6-Phosphogluconic acid	HMDB0001316	4.89	1.78E-04	-2.20
41	329.0309	Inosinic acid	HMDB0000175	5.51	1.97E-04	-2.10
42	333.2031	13-HODE	HMDB0004667	9.46	3.49E-03	2.16

43	335.0438	Nicotinic acid mononucleotide	HMDB0001132	4.33	9.46E-08	-2.31
44	346.9990	Inosine	HMDB0000195	6.72	1.76E-03	-1.53
45	349.1985	13-L-Hydroperoxylinoleic acid	HMDB0003871	9.36	2.49E-02	1.70
46	368.9881	dUDP	HMDB0001000	1.36	5.14E-03	1.77
47	369.0007	Sedoheptulose 1,7-bisphosphate	HMDB0060274	2.41	7.90E-05	1.93
48	374.0024	AICAR	HMDB0001517	1.41	4.23E-02	-1.63
49	379.2409	L-alpha-Acetyl-N-normethadol	METPA1411	9.63	1.86E-03	2.76
50	383.0494	Uridine 5'-monophosphate	HMDB0000288	5.96	4.98E-02	3.26
51	389.0470	1-Phosphatidyl-D-myo-inositol	-	5.10	5.66E-08	-2.05
52	407.1697	Thyrotropin releasing hormone	HMDB0060080	9.95	6.50E-06	16.72
53	433.0115	Diguanosine tetraphosphate	HMDB0001340	2.92	5.85E-03	1.78
54	464.9592	Inositol 1,3,4-trisphosphate	HMDB0001143	1.39	4.45E-06	3.19
55	481.2824	Campesterol	HMDB0002869	9.91	2.23E-02	5.16
56	493.3512	3a,7a,12a-Trihydroxy-5b-cholestan-26-al	HMDB0003533	9.75	5.28E-03	15.41
57	512.6592	Gamma-linolenoyl-CoA	HMDB0006368	1.28	4.18E-06	3.46
58	541.2595	Leukotriene D4	HMDB0003080	7.74	1.19E-02	-2.02
59	561.2482	Protoporphyrin IX	HMDB0000241	7.59	2.65E-02	7.42
60	568.6196	Chlordecone alcohol	HMDB0001219	1.29	6.22E-06	2.63
61	574.3066	Taurocholic acid	HMDB0000036	9.82	3.44E-02	2.08
62	586.2880	Psychosine sulfate	HMDB0013046	8.95	3.93E-02	-1.56
63	597.2353	Biliverdin	HMDB0001008	9.53	2.48E-02	2.92
64	666.0895	Uridine diphosphate-N-acetylglucosamine	HMDB0000290	1.51	1.63E-03	1.77

65	705.1134	Nicotinic acid adenine dinucleotide	HMDB0001179	2.39	7.70E-04	8.25
66	974.1316	Octanoyl-CoA	HMDB0001070	2.03	9.39E-03	-2.09
67	1040.1371	3-(4-methylpent-3-en-1-yl)pent-2-enedioyl-CoA	-	1.68	1.13E-02	-2.06

Supplementary Table S2: Altered metabolites list of Non-treated polycythemia vera vs treated polycythemia vera ($p<0.05$, Fold change >1.5)

Peak no	Mass to charge	**Metabolite**	**HMDB ID**	**Retention time (min)**	*p* value	**Fold Change (+,-)**
1	94.9235	Thiocyanate	HMDB0001453	1.39	6.29E-08	-2.15
2	96.9206	Hydrogen selenide	HMDB0011110	1.39	8.92E-09	-2.21
3	97.9588	Hydrogen carbonate	HMDB0000595	2.62	3.82E-04	-2.79
4	115.0398	alpha-Ketoisovaleric acid	HMDB0000019	1.59	4.29E-02	1.91
5	120.0115	L-Cysteine	HMDB0000574	6.62	2.28E-04	2.87
6	129.0187	Itaconic acid	HMDB0002092	1.52	4.36E-03	-2.00
7	132.0291	L-Aspartic acid	HMDB0000191	1.33	1.03E-04	-2.49
8	143.0792	Estriol	HMDB0000153	4.10	6.85E-05	2.06
9	157.0942	4-Hydroxyretinoic acid	HMDB0006254	4.78	1.72E-04	2.13
10	158.0449	Succinic acid	HMDB0000254	1.37	5.93E-03	-2.27
11	171.0760	5-Aminolevulinic acid	HMDB0001149	3.40	3.03E-03	1.78
12	187.0734	Glutamic acid	HMDB0000148	4.80	2.75E-02	1.53
13	188.0077	D-Xylitol	HMDB0002917	7.45	3.85E-02	-3.32
14	189.0854	(-)-trans-Carveol	HMDB0003450	4.46	4.71E-04	2.13
15	193.0241	1-Naphthaldehyde	HMDB0060325	1.51	6.71E-03	1.52
16	203.0008	Pyridoxal	HMDB0001545	8.19	1.08E-02	4.03
17	212.0234	3-Sulfinoalanine	HMDB0000996	2.63	1.01E-02	-2.28
18	215.0662	N-Acetyl-L-aspartic acid	HMDB0000812	5.32	2.67E-05	1.80
19	217.0289	D-Glucose	HMDB0000122	1.41	2.63E-09	-6.52
20	224.0570	L-Tryptophan	HMDB0000929	1.41	1.03E-02	-1.75
21	225.0747	Sinapyl alcohol	HMDB0013070	1.39	2.02E-05	3.31
22	507.2186	17-beta-Estradiol-3-glucuronide	HMDB0006224	1.18	1.97E-03	1.60
23	228.9626	alpha-Fluoro-beta-ureidopropionic acid	HMDB0060435	1.06	3.18E-03	2.11
24	232.9733	Nudifloramide	HMDB0004193	6.11	2.13E-02	1.51
25	233.0755	Formiminoglutamic acid	HMDB0000854	6.57	2.61E-02	2.66

26	237.0757	1,2-Dihydroxy-3,4-epoxy-1,2,3,4-tetrahydronaphthalene	HMDB0062273	7.94	4.85E-02	-2.57
27	239.0916	Coniferyl alcohol	HMDB0012915	5.96	2.30E-02	-2.72
28	264.1015	L-3-Hydroxykynurenine	HMDB0011631	1.41	1.74E-07	-3.25
29	268.7983	Iodine	HMDB0000675	1.34	1.71E-02	-2.13
30	269.0725	N2-Succinyl-L-ornithine	HMDB0001199	2.83	3.91E-05	1.96
31	273.0704	Deoxyuridine	HMDB0000012	4.50	2.22E-03	2.31
32	314.0648	D-4'-Phosphopantothenate	HMDB0001016	6.33	2.28E-05	2.27
33	315.0390	Nicotinamide ribotide	HMDB0000229	4.89	4.37E-09	3.39
34	316.0424	6-Phosphogluconic acid	HMDB0001316	5.51	7.33E-04	2.12
35	329.0309	Inosinic acid	HMDB0000175	4.33	1.96E-07	2.88
36	335.0438	Nicotinic acid mononucleotide	HMDB0001132	1.64	1.75E-02	2.17
37	343.0000	dCMP	HMDB0001202	4.23	1.38E-03	1.71
38	367.1558	Dehydroepiandrosterone sulfate	HMDB0001032	9.95	5.74E-03	4.21
39	348.9977	Inosine	HMDB0000195	9.76	3.03E-02	2.51
40	365.1952	Ubiquinol 8	HMDB0001060	1.36	6.49E-03	-1.94
41	368.9881	dUDP	HMDB0001000	1.41	6.48E-03	2.96
42	374.0024	AICAR	HMDB0001517	1.23	2.77E-03	1.66
43	403.0217	Phosphoribosyl formamidocarboxamide	HMDB0001439	9.95	6.77E-07	-10.06
44	407.1697	Thyrotropin releasing hormone	HMDB0060080	9.86	4.74E-03	-1.95
45	421.2197	Cortisol	HMDB0000063	9.90	1.05E-02	1.97
46	425.1176	11-Dehydrocorticosterone	HMDB0004029	2.92	7.24E-05	-2.68
47	433.0115	Diguanosine tetraphosphate	HMDB0001340	1.39	6.17E-03	-1.72
48	464.9592	Inositol 1,3,4-trisphosphate	HMDB0001143	9.17	1.89E-02	1.72
49	466.2504	7alpha-Hydroxy-3-oxo-4-cholestenoate	HMDB0012458	9.73	1.89E-02	2.62

50	483.1597	Estrone glucuronide	HMDB0004483	9.75	3.04E-03	-25.02
51	493.3512	3a,7a,12a-Trihydroxy-5b-cholestan-26-al	HMDB0003533	8.42	3.30E-02	1.72
52	512.6592	Gamma-linolenoyl-CoA	HMDB0006368	1.28	2.75E-02	-1.61
53	574.3066	Taurocholic acid	HMDB0000036	9.82	1.24E-02	-2.80
54	586.2880	Psychosine sulfate	HMDB0013046	8.95	3.54E-03	2.54
55	666.0895	Uridine diphosphate-N-acetylglucosamine	HMDB0000290	1.51	1.61E-02	-1.51
56	705.1134	Nicotinic acid adenine dinucleotide	HMDB0001179	2.39	1.28E-03	-6.37
57	875.5470	PS(16:0/16:0)	HMDB0000614	9.91	1.21E-03	-3.09
58	974.1316	Octanoyl-CoA	HMDB0001070	2.03	1.74E-03	4.51
59	1040.1371	3-(4-methylpent-3-en-1-yl)pent-2-enedioyl-CoA	-	1.68	6.47E-03	3.60
